# Letter from the Editor in Chief

**DOI:** 10.19102/icrm.2023.14126

**Published:** 2023-12-15

**Authors:** Moussa Mansour


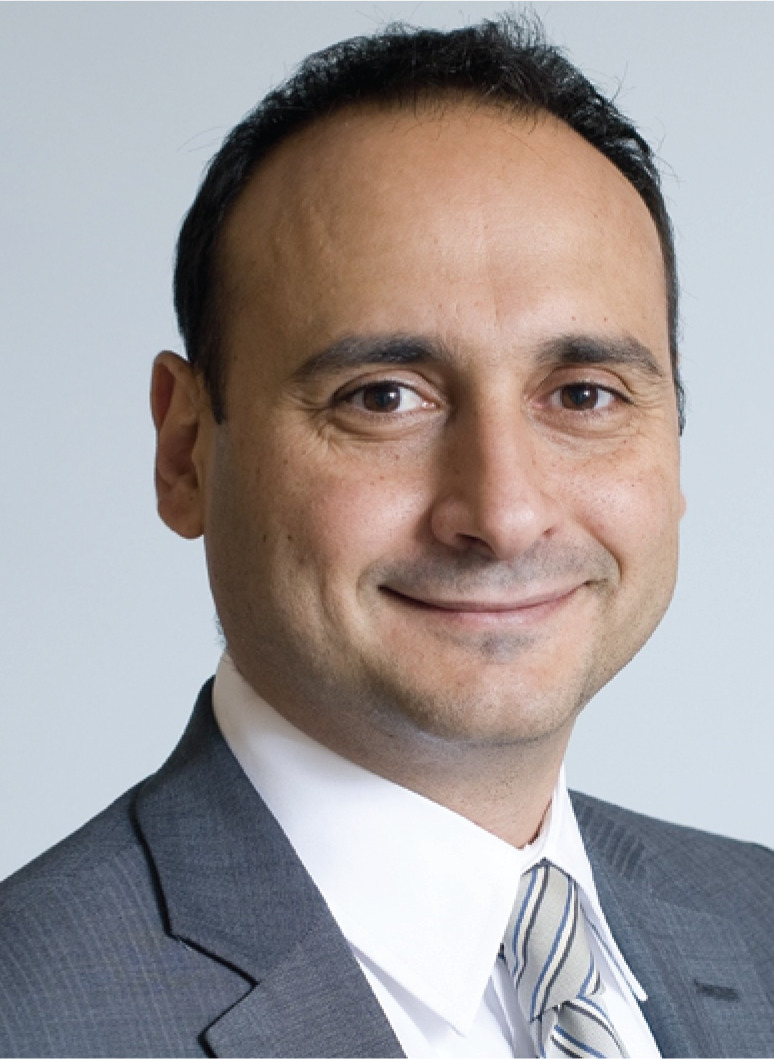


Dear readers,

This issue of *The Journal of Innovations in Cardiac Rhythm Management* contains many interesting articles. I would like to highlight the one by Sohinki et al. entitled “Pseudo-vagal Responses Elicited by Cryoballoon Ablation.”^1^ In it, the authors analyze the hemodynamic changes occurring during cryoballoon pulmonary vein (PV) isolation (PVI) and dispute the conventional account of vagal response as the only mechanism of hypotension. Instead, they provide an alternative explanation for the hypotension, consisting of vasodilatation secondary to the sudden release of cold PV blood into the systemic circulation. The study has some limitations, including a small number of patients, and its findings will need to be confirmed in larger studies. However, it is interesting because it highlights the role of autonomic denervation during catheter ablation for atrial fibrillation (AF).

Parasympathetic stimulation shortens the atrial refractory period, resulting in increased susceptibility to AF.^2,3^ The ganglionated plexi (GPs) containing the parasympathetic neurons are located in the pericardial fat near the ostia of the PVs.^3,4^ It has been generally accepted that concomitant autonomic denervation during PVI improves the success rate of AF ablation. However, the extent of autonomic denervation and its effect on AF recurrence after ablation appear to be energy-specific. Most of the early studies investigating the role of autonomic denervation during AF ablation used radiofrequency (RF) energy. At least one randomized study showed that the addition of GP ablation to PVI using RF improves the success rate of ablation compared to PVI alone.^5^ On the other hand, the additive benefit of autonomic denervation with cryoballoon PVI on the success rate of ablation AF outcomes is controversial, as discussed by Sohinki et al., with some studies showing that it can lower AF recurrence^6^ and others reporting no difference in outcome.^7^ More recently, the effect of pulsed field ablation (PFA) on autonomic modulation has been examined. A study measuring the increase in heart rate 3 months after ablation and using it as a sign for autonomic denervation found no effect with PFA compared to a significant effect with RF ablation and cryoablation.^3^ These results corroborated the findings of an earlier study showing that PFA compared to RF for PVI induces significantly weaker and less durable suppression of cardiac autonomic regulation when measured acutely during the ablation procedure.^8^ A third study using nerve-injury biomarkers as a surrogate for autonomic denervation showed that PFA spares the autonomic nervous system.^9^

PFA appears superior to thermal energy in almost every aspect, and it is expected to replace thermal energy as the primary energy source for PVI in the near future. It is unlikely that its lack of effect on autonomic modulation will change that course. However, studies will need to be conducted to investigate whether GP ablation continues to provide additional benefit. In that case, dual-energy systems allowing the delivery of both PF and RF may provide an advantage.

I hope that you enjoy reading this issue of *The Journal of Innovations in Cardiac Rhythm Management*.

Best wishes for a good 2024.


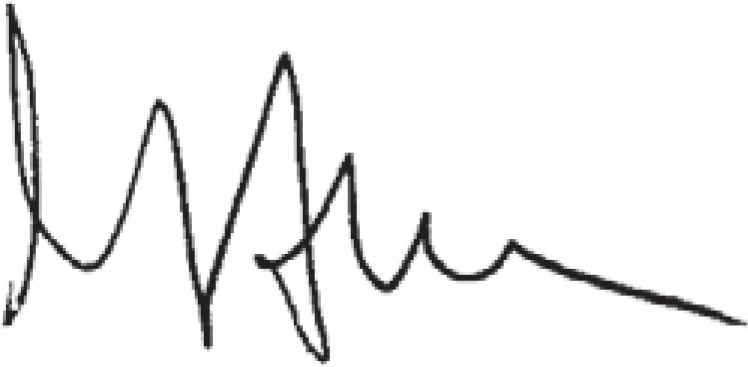


Sincerely,

Moussa Mansour, md, fhrs, facc

Editor in Chief

*The Journal of Innovations in Cardiac Rhythm Management*

MMansour@InnovationsInCRM.com

Director, Atrial Fibrillation Program

Jeremy Ruskin and Dan Starks Endowed Chair in Cardiology

Massachusetts General Hospital

Boston, MA 02114
